# Non-invasive 3D and 360° optical imaging of micro-particles

**DOI:** 10.1038/s41598-017-06830-8

**Published:** 2017-07-25

**Authors:** Toufic El Arnaout, P. J. Cullen

**Affiliations:** 1 PAT group, Dublin Institute of Technology, Dublin 1, Ireland; 2 School of Chemical Engineering, University of New South Wales, Sydney, Australia

## Abstract

Scanning electron microscopy and X-ray microtomography are useful methods for high resolution shape imaging. Visible microscopy is also common, however, developing a low-cost and customizable system for surface and shape investigation of optically active particles is challenging. In this work, we demonstrate an assembly offering good light sensitivity, flexibility of illumination and contrasts from varying angles. The design was applied, together with recent programs for focus-stacking, to analyze crystals of taurine, L-glutamic acid, acetylsalicylic acid, and copper sulfate, along with digital 3D-360° modelling of phosphorescent [Ru(bpy)_3_]Cl_2_ and strontium aluminate particles. We further tested the approach for real time monitoring of size, shape and texture analysis of fat filled milk particles and acid whey powders. The findings show proof of concept for detailed feature imaging of particles directly from the process environment.

## Introduction

### Pharmaceutical background and polymorphism

In 2004 the US Food and Drug Administration (FDA) released a guidance framework on Process Analytical Technology (PAT) for the pharmaceutical industry^1^, and discussed in other reports^2^ the importance of innovation at various production stages for good manufacturing practices, meeting process regulations and compliance with the Code of Federal Regulations (CFR) Title 21. The latter lists several regulations, requirements, and controls that govern the manufacture of drugs. Recently we developed optical PAT techniques for monitoring the crystallization process^3–6^, a critical step for active pharmaceutical ingredient (API) manufacturing, protein formulation (*e.g*., insulin) or structural determination by X-ray crystallography. Crystallization has different phases and often, polymorphs of varying size and shape distributions. Effective monitoring permits the control and optimization of the reaction process, feedback strategies, particle homogeneity, and process validation for batch consistency/signature. Pharmaceutical solid polymorphs constitute a wide field of research, and exhibit different physical and mechanical properties^7^: hygroscopicity, particle shape, density, flowability, and compactibility.

The characterization of particle surface features and 3D modelling could facilitate advanced process control for product quality investigation and process validation^8^. Such data could be applied in polymorph exploration and patenting for quality advantages and commercial competitiveness, and determining a crystal’s size-shape at high definition with implications on dissolution, bioavailability, shelf life, stability, etc. Recently, the guidance report by the FDA which related to industrial quality metrics^9^ insisted on the control and reproducibility of lot specifications. Such approaches are supportive of the proposed ‘Science-based’ lifecycle management and quality systems promoted by global regulatory agencies^10^ and in-line with the objectives of the ICH Q10 (International Conference on Harmonization) which promotes innovation, process technology, monitoring systems, and pharmaceutical quality, for continual product improvement^11^.

### Optical methods for crystallization monitoring

An in-line probe^4^ was previously fabricated by our group for the characterization of thin crystals and their surface features which was based on PlasDIC^12^, a simpler technique than the differential interference contrast (DIC) microscopy. However, the technique may be complex due to prism design and calculations, coupled with the fact that the different amplitudes and light intensities detected as contrast were generally relevant only for very thin and small particles. In order to study larger particles and a stronger contrast, a plan-apochromatic, stereo-microscope type objective was used, with flexible illumination angles, which enhances the micro-topographical observation.

Apochromatic objectives have been used in photography and telescopes (patents US2536508, 1951 and US3064532, 1962) for decades. Basic plan-apochromatic objectives have also been developed (*e.g*., patent US3756698, 1973). ‘Plan’ refers to the production of image surfaces that are flat for the field of view. Plan-achromatic objectives are constructed from conventional optical glasses, and usually have an in-axis chromatic aberration with an uneliminated secondary spectrum. This is corrected in plan-apochromatic assemblies, by employing a concave lens made of a crystal of alum, and a convex lens containing a crystal of fluorite. Both materials reduce the spherical aberration related to differences in color. Further developments reported in patent US3572902, 1971, employed a plan-apochromatic objective comprised of a forward lens group (six elements) and a rearward lens group (four elements), with fluorite only. Patent US3756698 described a 10X plan-apochromatic objective with two groups (four + two lenses). The secondary spectrum is corrected by a fluorite lens in the second group. By adjusting the dispersive powers of the other lenses (except the first), the system’s chromatic aberration is further corrected.

Today, plan-apochromatic objectives have corrections for a flat field of over 90% focus and for chromatic and spherical aberration for many colors. Their high resolution has resulted in their extensive use in machine vision applications. Configurations may vary to contain other materials (fluoro-crown glass, sapphire glass, synthetic quartz, diamond, liquids, or coatings like MgF_2_), and more lens elements to meet certain magnifications, numerical apertures, working distances, and specific applications. Today, achromats, fluorite/semi-apochromats, and apochromats are widely employed. The most recent apochromats are highly corrected and do not usually require additional correction through tube lens or eyepieces.

**Figure 1:**
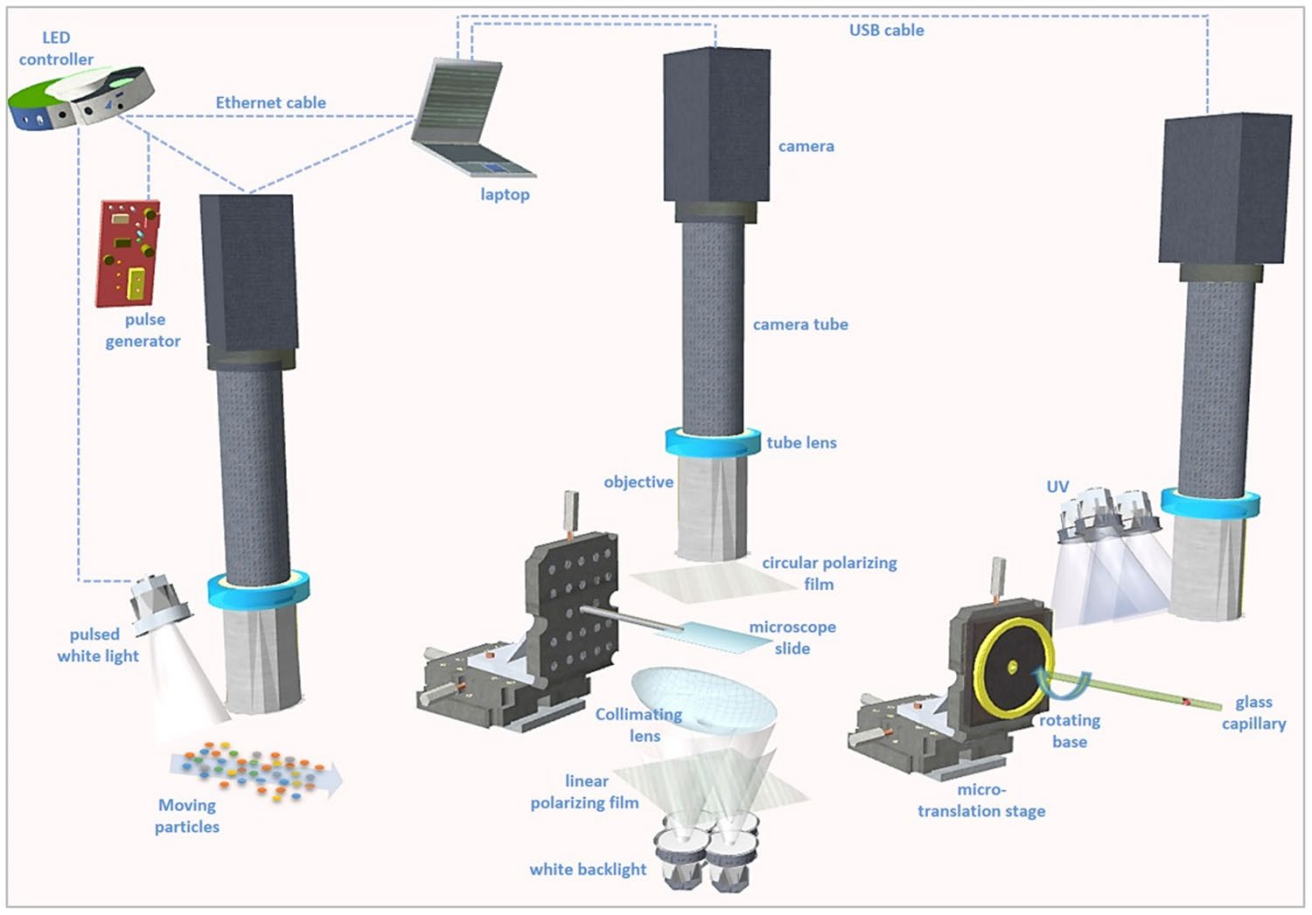
A 3D presentation of the setups in this study for real time size-shape monitoring, focus-stacking and 3D-360° photogrammetry of micro-particles. Left: the assembly can be used as a process analytical technology for imaging moving particles at high quality with pulsed camera and front-inclined white LED light. Middle: imaging of particles with white LED backlight with the xyz movement of particles for views from different angles (by rotating the particle or tilting the slide) as well as focus-stacking operations. The backlight collimating lens can be moved/rotated to adjust the backlight rays (*e.g*., condense, diffuse, direct, distribute, etc) depending on the optically active particle’s shape and features, particularly to optimize the surface contrast. Right: imaging of phosphorescent particles in UV. Particles inside a glass capillary tube (10 µm wall) are imaged at 360° for 3D modelling.

Owning to their good numerical aperture and light sensitivity at large working distances, plan-apochromats are consequently a good choice for applications requiring flexible illumination and 3D photogrammetry of sub-millimeter objects. In our design (Fig. 1) the approach provides a balanced depth of focus of 14 µm at a high resolution of ~2 µm and a practical field of view of 1.7 × 1.42 mm^2^ for imaging a range of sizes and counts. Several objectives were trialed in the selection of an optimum balance between magnification and image quality. For example, an objective of a 10X magnification usually has a depth of focus of 3–4 μm, and for a 20X, it is ~1.5 μm, which does not permit sufficient surface depths in focus and information in proximate pixels will contain higher blurriness and noise, particularly with optically active and complex morphology particles, resulting in low quality depth maps and 360° point coordinate images for constructing 3D models. Focus-stacking combined with 3D modelling is also difficult and introduces errors as shown later for a transparent crystal and sphere (Supplementary Figs 2 and 6). A natural image of the object, preserving its overall morphology at a large depth of field, as well as unique point match and overlap between images during rotation (*i.e*., low reflection, shiny spots, etc) is desired.

## Results

The assembly of the optical system presented in Fig. 1, provided a resolution at 5X magnification similar to that of expensive commercial systems, but with a higher practicality (*e.g*., larger field of view and depth of focus), and at a magnification up to 20–60 times lower. The resolution was found to be 1.44 pixels/µm, measured with a test target (Thorlabs, Cat. R1DS1P), with image dimensions of 2448 × 2048 pixels^2^. The R1DS1P test target has a maximum resolution of 228 line pairs/mm (*i.e*., 1 line pair = 6.34 pixels = 4.4 µm). Therefore the field of view is 1.7 × 1.42 mm^2^. The depth of focus is also excellent, considering that sometimes for stereomicroscopy it is in the mm range for a similar magnification but at a much weaker resolution. Furthermore, the numerical aperture for the field of view is remarkable given the 20 fold lower magnification. These points are most meaningful when compared to commercial systems of 10–40X eyepiece and 1–2X objective and the subsequent flexibility of the design.

**Figure 2:**
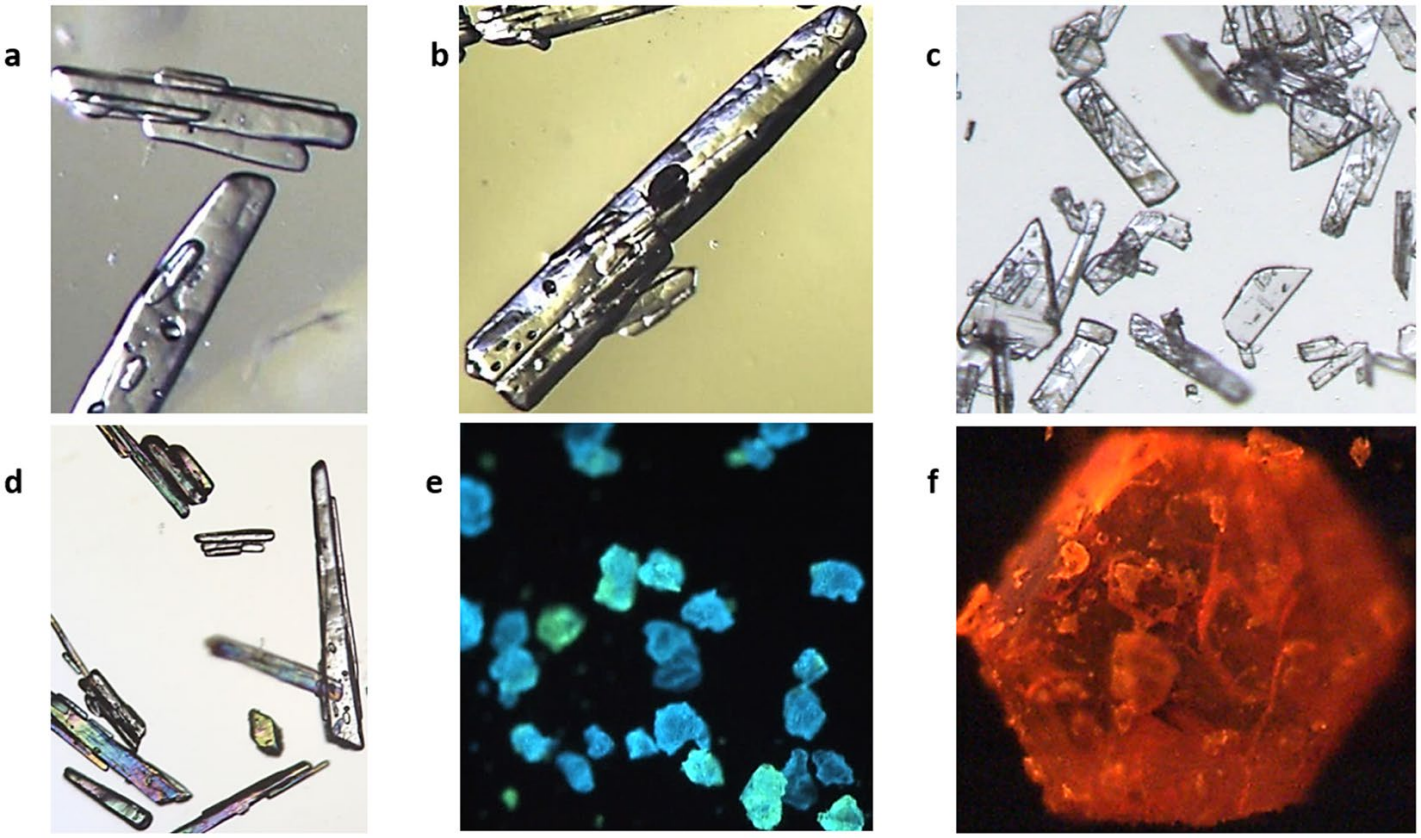
Particle imaging with a low-cost assembly for enhanced contrast and surface definition. Images in this figure do not constitute a focus-stack, and are snapshots cropped from larger field of view images (1.7 × 1.42 mm^2^). (**a** and **b**) Taurine crystals (360 and 850 µm, respectively). (**c**) Thin L-glutamic acid crystals (130 µm). (**d**) Taurine crystals (150 µm) viewed with cross polarizer films. (**e**) Strontium aluminate phosphorescent particles (80 µm). (**f**) Phosphorescent crystal of [Ru(bpy)_3_]Cl_2_ (440 µm). (**a**–**d**) were in white backlight in Fig. 1, middle, while (**e**–**f**) were taken in the dark using UV in Fig. 1, right, on a glass slide.

The field of view is suitable for analyzing crystals of common observed sizes (Fig. 2). The clarity and the illumination methods (darkfield, oblique, and brightfield) permit an enhanced contrast of microscopic 3D surface features that DIC methods have been known for^4^. However DIC may not be applicable for large particles and requires specific conditions and optical settings. In our case, thin crystals of taurine and glutamic acid could be visualized even with cross-polarized filters (Fig. 2d). With phosphorescent particles, such as granules of strontium aluminate or crystals of [Ru(bpy)_3_]Cl_2_ (chemical structure in Supplementary Fig. 3), the system in Fig. 1 (right) was beneficial mainly due to the advanced camera gain and shutter options.

**Figure 3:**
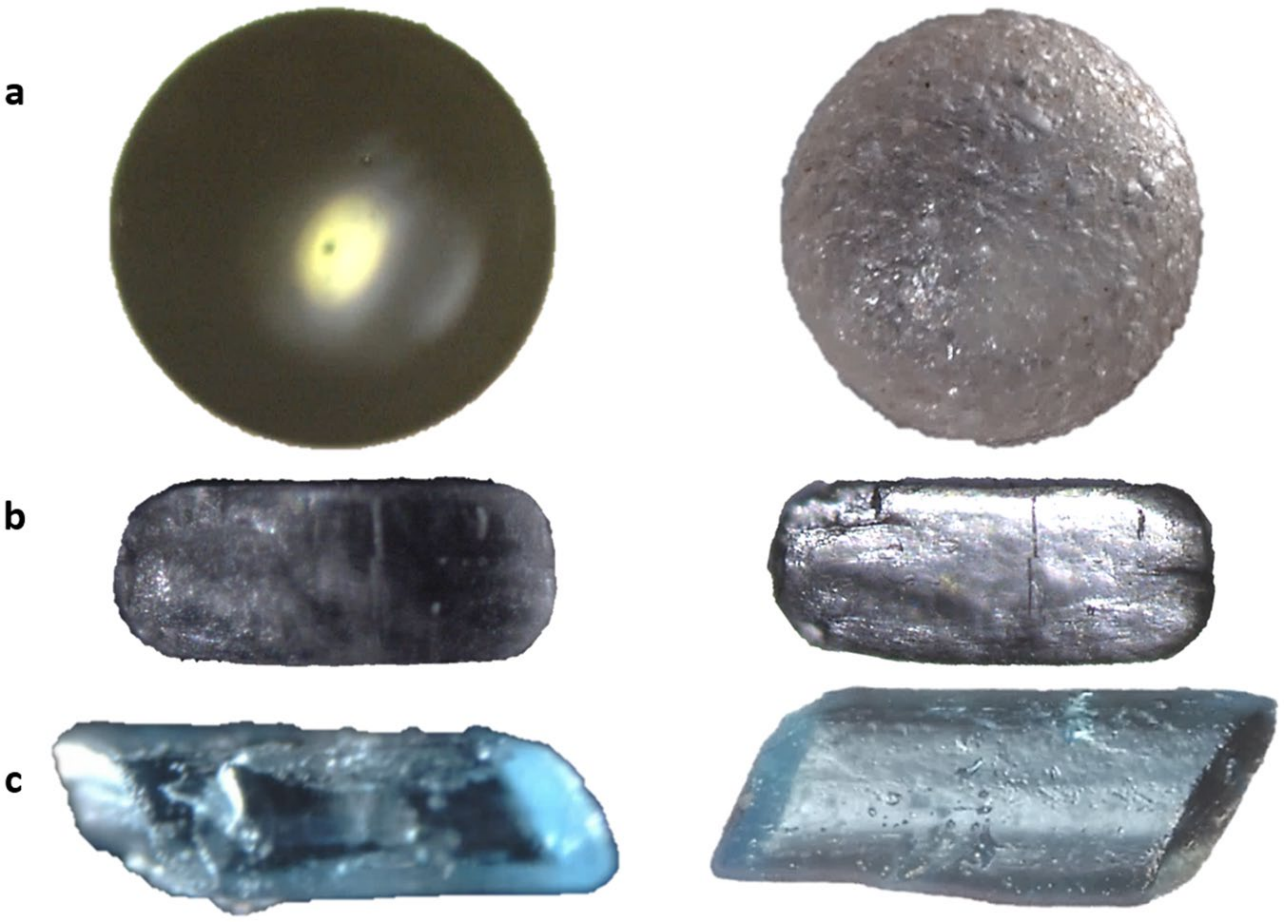
Imaging comparison between traditional microscopy and optimized optics. Traditional microscopy (left) does not have the illumination and setup optimized in respect of the optical activity of particles, compared to our method (right), which focuses on optimized background environment, and an innovative microscope objective combined with a specific illumination that permit the particle to self-distribute the light rays internally for better surface observation, rather than acting as a prism with uniform reflections. (**a**) A semi-transparent soda-lime glass sphere (700 µm). (**b**) An acetylsalicylic acid crystal (560 µm). (**c**) Different copper sulfate crystals (850 µm (left) and 950 µm (right)). Images of a, right, and c, right, were built based on focus-stacking. The other images are single snapshots. Images (to the right) were taken using the setup in Fig. 1, middle, with white backlight.

In oblique to darkfield modes, the illumination rays reach the particle’s plane at oblique angles with some reaching the objective as transmitted oblique illumination (Fig. 1, middle). In Fig. 1, left, light rays are reflected by particles, and in Fig. 1 right, particles self-illuminate by phosphorescence. The objective in our study has properties of enhanced contrast detection and light sensitivity, which can be enhanced through the light angle/incidence, diffusion or component lenses. This facilitates further diffusion of light internally within the particle rather than transmission of undesired focused refractions or direct reflections caused by their natural optical activity providing greater surface visibility. The setup in Fig. 1, middle, with white backlight permits these adjustments; the light source and collimating curved and large lens are tuned to optimized positions, according to each particle’s facet geometry and property (Fig. 3, and Supplementary Fig. 2). It is also crucial to control the environment behind the sample which is on the glass slide to avoid ambient rays from infinity reaching the objective, but mainly the crystal, which could cause unwanted internal reflections or transparency and blurriness. For this reason, images shown here would be difficult to obtain at a similar quality with a commercial stereomicroscope system particularly when a crystal is placed directly on an opaque dark surface with epi-illumination, or transmitted illumination when the particle is on top of the light source directly. Image examples of the impact of such classic methods of illumination can be seen in Fig. 3, left, and compared to our optimized setup. The depth of focus was generally practical, as seen for example in Fig. 2f, where surface texture features as low as 2 µm are detected on the 440 µm crystal of [Ru(bpy)_3_]Cl_2_. Conversely, observing the surface details for strontium aluminate (Fig. 2e) was challenging due to the high transparency and granular texture of the particles, probably due to the doping method and agent employed by the producer. Both of these chemicals are highly phosphorescent and can be chemically engineered; the chloride in [Ru(bpy)_3_]Cl_2_ can be replaced with anions such as hexafluorophosphate. Recently, [Ru(bpy)_3_]Cl_2_ was used as a photocatalyst with photochemical and redox properties, as well as an actinometer^13^.

**Figure 4:**
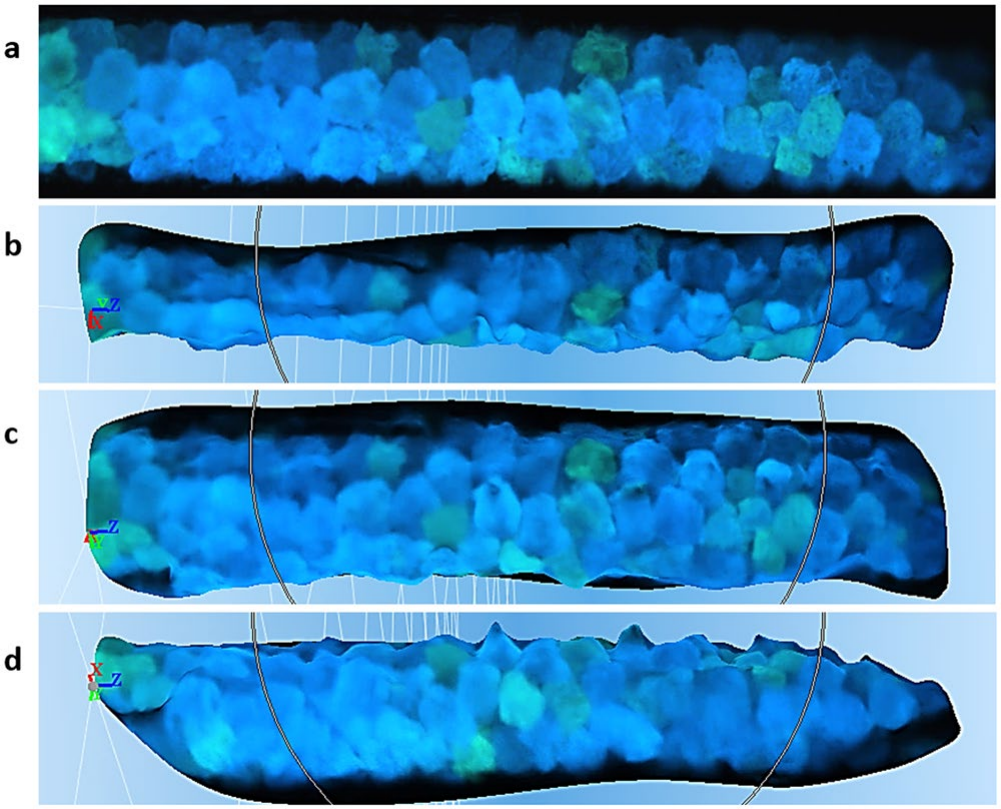
3D-360° photogrammetry for studying advanced shape and particle packing. Grains of strontium aluminate (60–90 µm) inside a soda-lime glass capillary tube (wall thickness: 10 µm; width: 300 µm; length: 1,550 µm) were imaged during semi-rotation. (**a**) Real image in UV in the dark, as described in Fig. 1, right. (**b**–**d**) 3D reconstructed model, viewed at −90°, −10° and +80°, respectively.

For 3D-360° image reconstruction by photogrammetry, we imaged an engineered sample of phosphorescent strontium aluminate particles in a capillary tube (Fig. 1, right). 3D photogrammetry programs require acceptable surface markers and reference points between images, facilitated here through auto-illumination and the presence of multiple blue or green granules (Fig. 4). Packing and geometry were observed along the tube and 3D shape and density can be determined.

**Figure 5:**
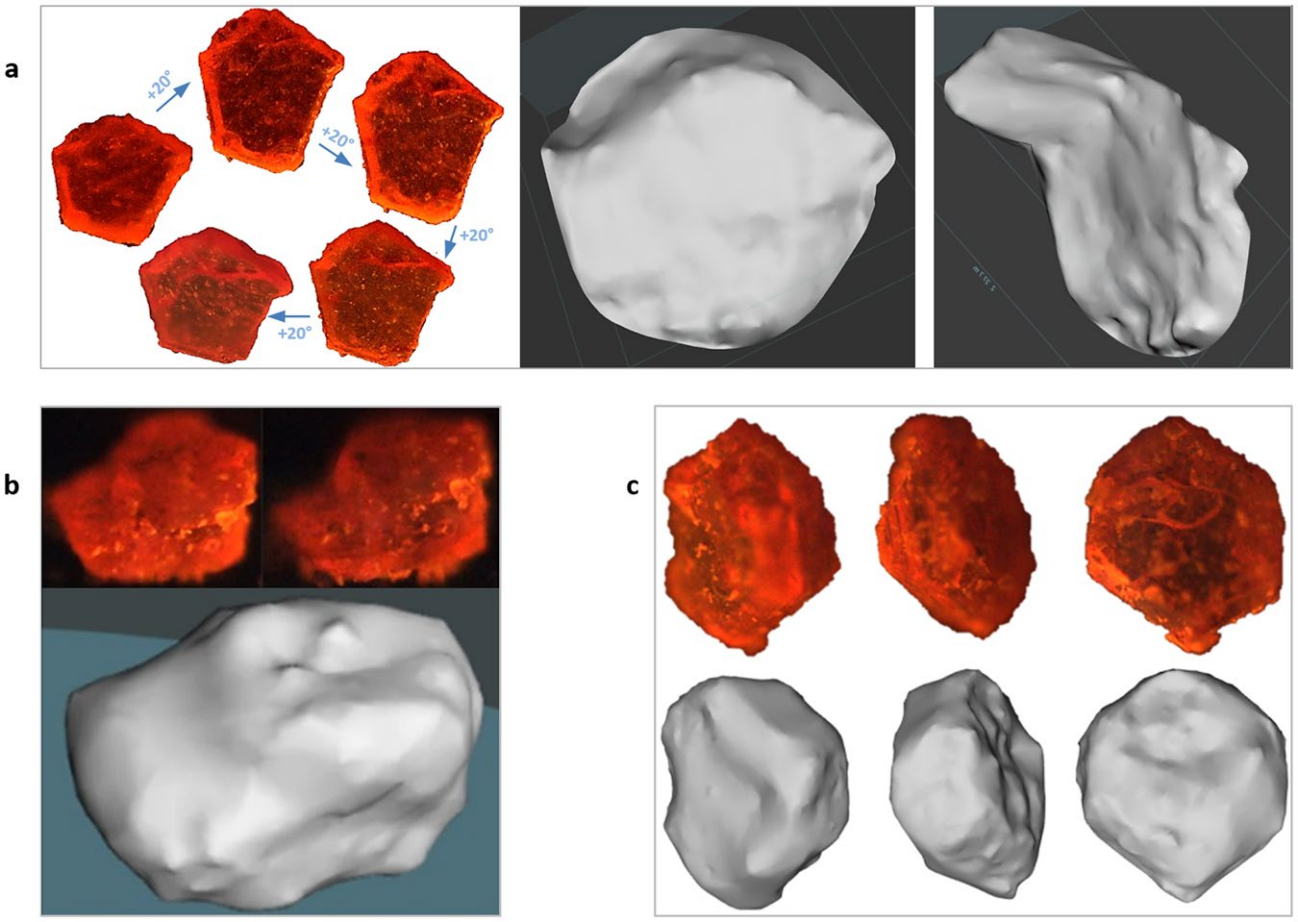
Full 3D-360° digital reconstruction of a single micro-particle. [Ru(bpy)_3_]Cl_2_ crystals were imaged in the dark as in Fig. 1, right, to allow auto-illumination by phosphorescence. (**a**) Ultra-thin solid flake (length: 540 µm). Left (**a**): Real images from −40° to +40°. Right (**a**): Views from top and side of the digital surface reconstruction. (**b**) Real images (top) of a second particle (285 µm) and the digital 3D model (bottom). (**c**) Real images (top) of a third particle (440 µm) and the digital 3D model (bottom).

The next step was single particle 3D-360° analysis of [Ru(bpy)_3_]Cl_2_ crystals (Fig. 5). Larger particles, while requiring high resolution imaging, are challenging due to out-of-focus domains within the same image and during 360° acquisition. Several 3D reconstruction programs may produce different results, such as Agisoft PhotoScan, Reality capture, 3DF Zephyr, Autodesk ReMake, Autodesk 123D Catch, and VisualSFM.

The flake particle’s model (Fig. 5a) reveals the depths and curvatures that cannot be seen in a single real image. For a smaller particle (150 × 200 × 285 µm^3^), the 360° rotation permitted complete 3D reconstruction despite the variety of zones in focus related to the particle’s shape (Fig. 5b). It was also possible to build the 3D shape of a larger particle with surface markers (Fig. 5c), with clear geometrical details of individual facets.

**Figure 6:**
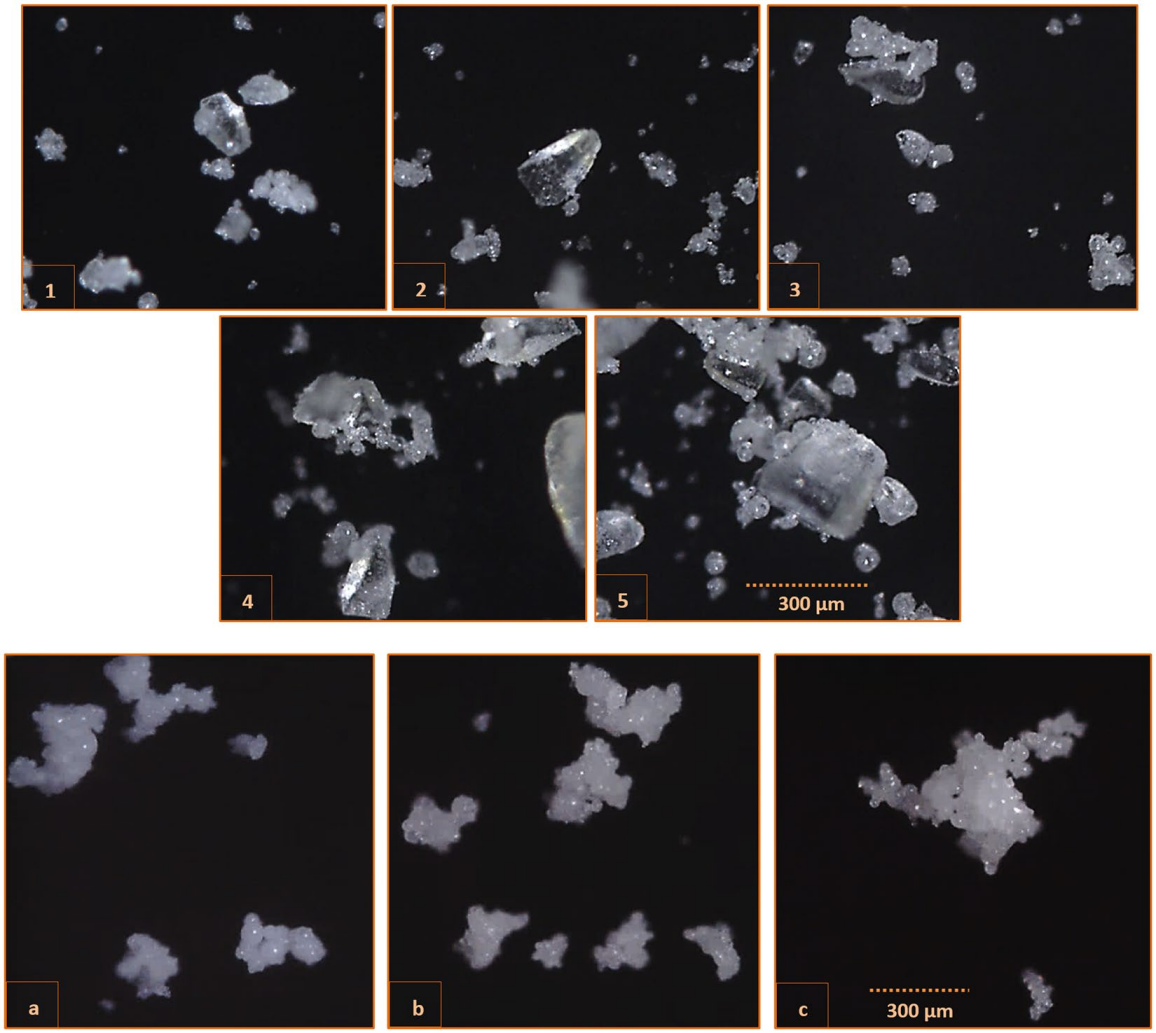
High speed imaging of milk derivative product particles. 1–5: Samples of acid whey powder with poor solubility. **a**–**c** Samples of fat filled milk. Images were taken on a moving sample holder using the setup in Fig. 1, left, with pulsed white light allowing real time monitoring. Image processing results and statistics of particle size-shape distributions are shown in Table 1.

In the last application, we tested the design for analysis of milk-derivative samples (Fig. 6) with reflected pulsed camera/white light as described in Fig. 1, left. The design offers the potential for real-time particle image analysis. The acid whey samples were of poor solubility (Figs 1–6) and contained α-lactose crystals of a typical tomahawk/prismatic shape. The transparency of the crystals can be also evaluated with the size-shape distribution, accomplished here via imaging algorithms. The difference in texture and creamy tan is clear between samples 1–5 and the three fat filled milk powder (FFMP) samples a-c, likely occurring due to the composition, processing, and coatings during blending (*e.g*., hardened palm oil, palm oil, coconut oil, etc).

Size-shape distributions were determined (Table 1) by analysis of the resulting images (Macro in Supplementary material) which generally contained separated individual particles, to avoid errors associated with the watershed algorithms at this stage. Size here refers to feret’s diameter (distance of maximum caliper). The average is based on all images obtained for a sample. The shape descriptors are the aspect ratio, equal to the major axis/minor axis of the fitted ellipse, the circularity, equal to 4π × Area/Perimeter^2^, and the solidity, equal to the Area/Convex Area.

**Table 1:** Size-shape distribution of acid whey powder and fat filled milk samples based on image analysis.

Sample	Average size (µm)	Average circularity	Average solidity	Insolubility	Dn_10_ (µm)	Dn_50_ (µm)	Dn_90_ (µm)											
1	85.5	0.8	0.93	0.2	15.4	47.4	198.7											
2	79.3	0.81	0.94	1.2	16.4	44.2	167.9											
3	79.5	0.8	0.94	1.9	17.1	47.6	178.1											
4	76.8	0.8	0.93	3	16.2	46.9	164.1											
5	76.5	0.8	0.93	4.5	15.4	45.8	152.8											
				**Distribution (%)**														
				**Size (µm)**						**Circularity**			**Solidity**			**Aspect ratio**		
				0–20	20–50	50–100	100–250	250–500	500–1000	0–0.75	0.75–0.9	0.9–1	0–0.92	0.92–0.96	0.96–1	1–1.5	1.5–2	2–3.5
a	304.7	0.56	0.85	3	9	8	36	30	14	80	14	6	74	20	6	55	35	11
b	284.8	0.58	0.86	2	1	7	51	29	9	80	18	2	72	16	12	41	46	13
c	347.4	0.55	0.85	2	9	6	28	36	19	81	15	4	74	17	9	43	32	23

1–5: Samples of acid whey powder with poor solubility. a–c: Samples of fat filled milk (FFMP). Average values are based on all particles for each sample. Distribution is based on the counts within an interval, compared to the total data array for a specific descriptor. Dn is based on number/count weighted distribution of particles, and not volume (Dv), for cumulative percentages 10, 50 and 90.

For samples 1–5, the determined average size was proportional to the insolubility factor from 0.2 to 4.5. Dn_90_ also decreased proportionally. However, circularity and solidity remained stable. For the FFMP (samples a-c), the average size was 3–5 times higher than that of the acid whey powder samples (1–5). The circularity was much lower, as well as the convexity/solidity factor which indicates less regular edges.

Sample b had most of its distribution in the 100–250 µm range, ~50% more than samples a and c, but less in counts for the high range of 500–1000 µm. For shape, particles of circularity 0.75–1 and solidity 0.92–1 were probably those mostly affected by the filling type of fat. Particles of aspect ratio 1–1.5 likely increased towards 1.5–2 in sample b, compared to a, but towards 2–3.5 in sample c, compared to a.

## Discussion

### Optical assembly

The assemblies in Fig. 1 offer the potential for process monitoring, focus-stack imaging, as well as 3D-360° digital reconstruction of particles. The large collimating lens (Fig. 1, middle) can be moved and rotated freely to modify the rays of ‘white backlight’ (e.g., diffusion, divergence, condensation, deflection, negative/positive relief). This permits rapid optimization of the observation and surface contrast depending on the particle’s size-shape and optical features (Fig. 2), and minimizes the collection of rays due to optical activity of the crystal. Therefore, phase structures and not simply amplitudes are detected (Fig. 3), improving focus-stacking at high resolutions. The ambient background settings behind the crystal are also critical, because an open atmosphere introduces internal particle reflections and transparency. We maintained a dark environment around the system and behind the light source, with a balance of light to sample intensity and distance. Furthermore, the objective’s numerical aperture and camera sensor settings of gain and shutter permit the effective amplification of recorded signals (Fig. 3, right).

### Detection of surface characteristics

Employing a macro-lens for enhanced contrast and resolution, facilitated detection of detailed surface features within a practical depth of focus, or for the entire particle through focus-stacking. For example, we imaged acetylsalicylic acid without focus-stacking (Fig. 3b), but for thicker particles with stronger edges and surface features, focus-stacking can be performed (Supplementary Figs 1 and 2). Particle features such as a 2 µm wide fracture in an acetylsalicylic acid particle (Fig. 3b), and microscopic pores of 2–4 µm diameter on the surface of a copper sulfate crystal (Fig. 3c) were visible. It may be also possible to extend the application to study micro-width fluidic grooves^14^. Therefore, the system has potential in materials science studies of micro-cracks and micro-voids, as well as for example surface and shape characteristics and control of ALiCE (activated lignin-chitosan extruded) pellets^15^. The design could replace scanning and transmission electron microscopy (TEM) for certain applications such as imaging microstructures and damages (5–20 µm) of aluminum bronze (QAL9–4)^16^ possibly caused by the dislocation of the crystal lattice and formation of re-crystallization grains inside an adiabatic shear band. Furthermore, microparticles (*e.g*., fine particles) adhered onto larger ones were visible, which is impossible with chord length/laser based methods. We presented an example for [Ru(bpy)_3_]Cl_2_ during phosphorescence (Fig. 2f), and another with focus-stacking (Supplementary Fig. 3).

### 3D photogrammetry of particles

The optimized illumination was further enhanced with a linear polarizing film for the light source and a circular polarizing film for the objective. However, it remained challenging to build 360° digital models using the setup in Fig. 1, middle. Supplementary Fig. 2 explains the difficulty for a copper sulfate crystal where focus-stackings were performed for 25° step rotations, where edges and facets did not constitute matching markers or reference points from the different angles of view. Determining depth maps from the focus-stacks was also difficult, due to some remaining transparencies and reflections. Such reflections were minimized, however facets remained, appearing differently due to the varying particle’s thickness and shape in specific directions, influencing the internal light distribution and transmitted rays, while changing the angle of observation and/or position of the crystal. Additional light sources tested at other angles caused further transparencies, loss of surface of interest contrast, and reflection spots.

An example of a successful 3D photogrammetry of a particle not using the self-illumination by phosphorescence, but by the normal backlight outlined in Fig. 1, middle, is demonstrated for the sphere in Fig. 3a, and in Supplementary Fig. 6. The result was based on feeding the program a total of 900–1,200 images representing a total of 20 angles of view (45–60 focal plane snapshots each). The main limitation in this experiment, apart from the large variety of in-focus and out-of-focus pixels, is the geometrical symmetry of the particle, posing a challenge which often occurs with 3D photogrammetry programs, due to the machine’s limitation in image understanding. For example, a cube is seen identically from the different peaks formed by the repetitive vertex/edge geometrical arrangements, which causes a challenge for machines. As a result, the calculated model is not a full sphere but a semi-sphere, with the remaining half represented by points placed behind the dome into infinity, forming a paraboloid, and some points placed inside the calculated model probably due to the pixels from the different focal plane images at different angles. A semi-sphere result is nevertheless promising because most 3D programs tested were not successful in this experiment. For other shapes and particles, programs may fail to “close” a model over 360° as shown in Supplementary Fig. 4. A secondary observation in Supplementary Fig. 6 is the curvature of the background’s constructed plane, probably caused by the Plan-apochromatic objective.

Programs of 3D photogrammetry are constantly being improved, nevertheless, several systems require additional programs for dense cloud and mesh operations, texture fitting, refinement and measurements, etc. In addition, images in a dataset have to be chosen carefully, as particular images may be the reason of significant errors in the model or even the rejection of the entire dataset.

### Advantages of the current technique

The illumination is flexible, allowing full rotation and movement of components along with addition of films or lenses to enhance the contrast and phases based on the particle’s texture, curvature, shape, or size. The sample holder is also movable (Fig. 1, middle and right). This method may help with other studies of surface details, phase separation or organization of particles such as aqueous suspensions of cholesteric cellulose nanocrystals (CNCs) (~100–150 µm)^17^, with polarized films that functioned for taurine crystals here (Fig. 2d). The modifications permit the collection of optimized field illumination of the particle and reduce signals of transparency, reflections or undesired optical activity of crystals. Imaging and sensitivity were balanced due to the camera performance and the objective’s numerical aperture for high resolution, a practical depth of focus and field of view coupled with long working distances for a narrower vertical conical angle (sample to objective) signifying the collection of more specific light rays. Optical correction due to the sample holder was also not necessary, as typically the glass slide introduces chromatic and spherical aberration in some applications, negatively impacting the contrast and requiring extra configurations for direct sample imaging. This advantage may contribute to a consistency in imaging quality during all stages of *in situ* crystallization monitoring^3, 4^ facilitating tolerance of process variations in concentration, temperature, particle thickness, and solution.

Texture analysis of milk derived particles was also demonstrated, with images compatible with the processing algorithms for determining size-shape distributions (Fig. 6 and Table 1). Rapidly moving particles imaged using a Process Analytical Technology (PAT) based principle for real time monitoring purposes is also possible, and further systems could be adapted and customized for monitoring particles inside chambers, spray dryers, blenders, or through a glass window with moving particles illuminated and imaged via the pulsed global shutter camera and flexible LED.

Imaging of sub-millimeter, semi-transparent particles that emit light by phosphorescence in the UV was also straight-forward, and the particle’s profile (Fig. 5) appeared to be less affected by its thickness and transparency, or angle with the light source and objective, than for the crystals in visible light such as copper sulfate (Supplementary Fig. 2), which simplifies 3D photogrammetry applications. Furthermore, operating in UV light may enable tracking of complex optofluidic fabrication of 3D-shaped polymer particles^18^, or at different wavelengths for the investigation of zeolite ZSM-5 crystals (20 × 20 × 100 μm^3^)^19^. In material science, graphene flakes could also be investigated, in addition to membranes for nanoparticles that are micro-engineered based on specific pore morphology, arrangement, and size^20^.

### Current challenges: from particle properties to algorithms

Even though the contrast of surface features was enhanced, it was difficult to construct 3D-360° transparent particle models (Supplementary Fig. 6). This is due to the contrast and light transmission dependent on the particle’s light transmission and distribution changing during rotation as observed for the same facet (Supplementary Fig. 2), and also case-dependent on the particle’s shape, thickness and position between the light beam and the objective (Fig. 3b and c). Building a 3D model of a symmetrical object is also difficult (Supplementary Fig. 6) for the program because of the presence of remaining reflection spots at the same coordinates at every rotation. In addition, apart from the stacking quality, focus-stacked imaging of large particles is challenging for the 3D photogrammetry programs, and single snapshot images of large depths of focus are preferred.

Another challenge is that the camera usually rotates around a fixed object in an environment and not the opposite. However, in our setup (Fig. 1, middle and right) in a dark background with a phosphorescent particle, both methods of recording (camera around sample or sample rotating under the camera) produce identical images. Programs may consequently build a surface that is a mirror of the reality, or with inverted curves for certain facets and domains, or may fully/partially close the model in the wrong direction (Supplementary Fig. 4 compared with Figs 4 and 5). Other program issues include failure to interpret some calculated camera positions correctly during dense cloud generation (Supplementary Fig. 5), which often limited the construction to 180° instead of 360°, particularly due to in-focus/out-of-focus domains, and for objects of high symmetry, weak surface markers, insufficient overlapping references between images, and those of strong transparency, translucency, shiny spots and reflections. Crystals are particularly challenging, due to their specular smooth surfaces, anisotropy, and optical activity. It is hoped that advancements in 3D photogrammetry, such as overcoming transparent object surfaces in certain situations^21^ through novel visual hull refinement schemes and contour tracking, can be incorporated. In our current study, with imaged angles for self-illuminating particles, 3D models were remarkable for strontium aluminate and [Ru(bpy)_3_]Cl_2_ (Figs 4 and 5).

### Comparison with other methods: flexibility and limitations

The low-cost and simplicity of this non-invasive approach offer significant advantages for particle imaging including; flexibility, high resolution and suitability for surface analysis, 3D studies, and possibly shape analysis of protein microcrystals for different applications^22, 23^. Although scanning electron microscopy (SEM) images provide exceptional signal to noise ratio, the sample’s electrostatic charging is commonly encountered at high voltage and such approaches are not compatible for process applications. Sophisticated detectors, labeling molecules or secondary phase nanoparticles were also not required with this design.

Historically with SEM, the term 3D photogrammetry in the 1970s-80s generally meant basic topographic models of the surface by 2–3 tilting exposure images at different angles^24^. For 3D modelling with 360° SEM, the approach requires advanced calibration, with an understanding of key parameters; object’s tilt and positioning, eucentric axis/rotation, and the detector’s settings. Labeling, control points and optimization algorithms can be also challenging. The advantage of this method is a ~20 times higher resolution than that of most macro-type objectives^25^.

In more recent topography studies, SEM data was combined with confocal laser scanning microscopy^26^, to produce 3D models of diatom frustules (130 μm), coated with a gold/palladium layer (20 nm). Two eucentric tilted images were taken (−5° and +5°) to construct the surface model. Other studies applied transmission electron tomography on poly(L-lactic acid) (PLLA) crystalsomes (~0.5 µm)^27^, with limitations in terms of crystallographic directions that can be detected and for sizes <0.5 µm. Only very recently with SEM and 3D photogrammetry, the full 3D shape of a ~6.5 µm particle has been reported^28^.

X-ray microtomography (μCT) is another method for 3D analysis, available in a benchtop imaging instrument^29^ and at synchrotrons. The spot size is ~5 µm but recently improved > 5–10 times due to a higher efficiency in the focusing and zone plates, leading to the method being termed nano-CT^30^. μCT was applied with computing to quantitatively assess the 3D shape of particles of 100s of µm up to 2–3 mm^31^. Synchrotron radiation (SR-μCT) assisted with imaging the polymorphism of clopidogrel bisulphate (CLP) and the 3D distribution profile of particles (generally 300–600 µm) within capsules^32^. Samples were scanned at 16 KeV over 180° to record 720 projection images.

## Conclusion

It was possible to observe fractures, porosity, surface markers, and microstructures of particles of different transparency levels, as well as build focus-stacks and 3D-360° models for several particle types, with a dedicated optical system and illumination. It is hoped that future algorithms in 3D photogrammetry will be capable of assigning advanced reference points when working with several particles in a sample, and analyze texture, roughness and polymorphism. Currently, basic geometrical measurements from the 3D mesh are possible, such as volume, surface area, center of mass, symmetry, etc.

Developing automated scripts that combine and optimize focus-stacking with 3D photogrammetry, for dedicated conditions is recommended. Developments from other fields are encouraged, including improved detectors, light sheet surface illumination, use of micro-markers and programming with image understanding, for additional optimization in the 3D modeling of challenging particles, and to overcome difficulties related to the optical and focal limitations.

The crystal imaging examples and tools presented here could also be useful for other applications such as creating advanced population balance models (PBM) based not only on chord measurement (1D) or length and width (2D)^33^ of particles, but on full 3D shapes for more accurate simulations and kinetics, for batch cooling crystallization processes^34^ and continuous manufacturing employing a PAT strategy^35^. Other possible examples include enhanced contact shape visualization of droplet-surface interactions^36^, advanced visualization of engineered particles in fluidized-beds, and crystal shape and positioning inside mounted loops at synchrotron beamlines for X-ray crystallography^37^ aiding in rastering techniques^38^.

Finally, the electronics-optics-algorithms representation of a process analytical technology method in this study, based on the LED controller for pulsed camera/LED for imaging moving particles in real time is important for process monitoring and determining size-shape in different situations and industries.

## Methods

### Imaging setup

A schematic of the overall experimental design is shown in Fig. 1. The camera is a C-mount (Point Grey, Cat. GS3-U3–50S5C-C) equipped with a Sony ICX625 CCD, 5 MP (2448 × 2048), global shutter, 2/3″, pixel size 3.45 µm, and maximum 15 FPS. A 152.5 mm tube was connected to the camera (Edmund optics, Cat. 56–992) from one side and to the 1X tube lens (Edmund optics, Cat. 54–428) from the other. The objective (Mitutoyo) is a M Plan Apo (Edmund optics, Cat. 46–143): magnification 5X, infinity corrected, numerical aperture 0.14, working distance 34 mm, focal length 40 mm, resolving power ~2 µm, depth of focus 14 µm, diameter 34 mm, and corrected for 435–655 nm. For visible light illumination of crystal or milk particles, a strong LED light source was used as a backlight (Fig. 1, middle), or front-inclined pulsed mode (Fig. 1, left), respectively, while for 3D photogrammetry in UV (Fig. 1, right), the lamp’s (5 × 15 cm^2^) wavelength was 365 nm. For the visible backlight application with focus-stacking of transparent crystals and for building the sphere’s 3D model, a linear polarizing film was added to the light source while a circular polarizing film added to the objective (slightly change the objective’s working distance). Between the white backlight source and the sample, a large (~4 cm) collimating lens was placed, allowing the modification of direction, angle, relief contrast, and amount of light rays, depending on its position and rotation. The micro-translation of the sample was possible by a xyz stage, with an arm for the glass slide rotatable to a certain degree depending on the particle’s stability. The arm can be removed for fixing a 360° rotating base, manually glued to a soda lime glass capillary (Hampton Research, Cat HR6–156, diameter 300 µm, wall thickness 10 µm, with a conical opening for mounting particle) for full rotation.

### Imaging settings

The camera’s software (Flycapture 2.9.3.43) was used for adjusting the settings and recording in both manual or automated modes, of the camera connected to a laptop via USB 3.0. For visible light imaging (*e.g*., glass sphere, copper sulfate crystal, etc), the camera settings were: shutter 40 ms, gain 15 dB, brightness 1.42% and gamma 1.76. For phosphorescence images (*e.g*., strontium aluminate, [Ru(bpy)_3_]Cl_2_, etc): shutter 165 ms, gain 18 dB, brightness 0.62% and gamma 0.78. For moving particles, the strobe settings (Fig. 1, left) can be adjusted via a programmed LED controller and camera’s software, connected with a pulse generator for additional protection (in average 2 fps), with fast shutter/LED pulses and average gain (10–15 dB), with continuous image recording via the Flycapture software, and processing algorithms (Supplementary material) to determine size-shape and statistics.

### Particles

All chemicals were purchased from Sigma-Aldrich: blue-green phosphor strontium aluminate, europium and dysprosium doped (Sr_4_Al_14_O_25_: Eu, Dy) (Cat 756520); red crystalline phosphorescent salt of [Ru(bpy)_3_]Cl_2_ (tris(2,2′-bipyridyl)dichlororuthenium hexahydrate) (C_30_H_24_Cl_2_N_6_Ru · 6H_2_O) (Cat 224758); L-glutamic acid (Cat. W328502); taurine (Cat. W381306); acetylsalicylic acid (Cat. A5376); and copper sulfate pentahydrate (Cat. 61245). The semi-transparent glass sphere was picked from a polydisperse size mixture (LGC standards, Cat. WS-PS237). The dairy samples were shown for demonstration of the application only, and were obtained from collaborators: five samples of poor solubility acid whey powder (samples 1–5 with pre-determined insolubility factors), and three samples of fat filled milk powder (samples a-c).

### Focus-stacking

Images (*e.g*., 50–100) were first imported to Picolay (version 2016–05–26, Heribert Cypionka, www.picolay.de). The first step was to auto-align the positions and resize, saving the images separately rather than directly performing stacking with auto-alignment activated. The optimized images were then imported to a blank session, and stacking parameters were used by default: smart filter activated and minimum contrast set to 0.

### 3D photogrammetry

3D reconstruction of strontium aluminate particles in the capillary was carried out in Autodesk 123D Catch. For [Ru(bpy)_3_]Cl_2_ crystals, Autodesk ReMake was used. For the sphere reconstruction shown in Supplementary Fig. 6, Agisoft PhotoScan was employed. After importing or uploading the photos to the photogrammetry software or server (depending on the company), the first step is alignment of photos in order to identify matches, geometries, and camera positions, and build a sparse point cloud. A dense cloud is then built and pixels are allocated their spatial locations. Manual cleaning is possible to remove erroneous, out of shape, or unwanted points. A polygon mesh can be constructed, connecting for example a group of points into triangular fragments. The surface of the model is based on a complete mesh from all of those fragment groups. Additional steps are possible, such as gap bridging, surface and boundary smoothing, or calculating a texture fit based on the original images (as in Supplementary Fig. 4). The 3D model also permits geometrical calculations.

### Image analysis for size-shape of dairy particles

Particles were placed onto a glass slide, and 30 random images were recorded per sample during motion with particles in focus and with minimal touching/overlap, to avoid the watershed/segmentation step at this stage in order to minimize their error related to the presence of random shapes. Batch image processing was carried out in ImageJ^39^, starting with contrast enhancement by linear contrast stretching. Other functions: smoothing filter, Despeckle function, outlier removal tool, dilation, and filling holes were employed. Measurements were performed for the area, perimeter, shape, feret, circularity, solidity, aspect ratio, etc. Particle size and shape distributions and Dn values were calculated in Microsoft Excel. The Macro text of the functions employed is shown in the Supplementary material.

## Electronic supplementary material

Supplementary information
