# Supplementary material for: Non-invasive 3D and 360° optical imaging of micro-particles
Source: Sci Rep. 2017 Jul 25;7:6384. doi: 10.1038/s41598-017-06830-8 (PMC5527111; doi:10.1038/s41598-017-06830-8)
Supplement: Supplementary file 1 — Supplementary information [file 41598_2017_6830_MOESM1_ESM.pdf]

Non-invasive 3D and 360° optical imaging of micro-particles

Toufic El Arnaout, P.J. Cullen

Supplementary material

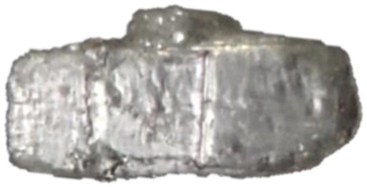

Supplementary figure 1: Focus-stack image of an acetylsalicylic acid particle. Length: 680 µm.

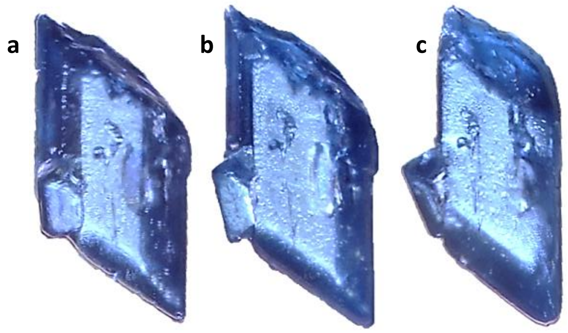

Supplementary figure 2: Focus-stack images of a copper sulfate thick crystal for different angles of view. a: Rotation by +25° of particle in "b". b: 0° initial position. c: Rotation by -25° of particle in "b". The maximum length of the crystal is 760 µm. Each focus-stacks was based on ~70 images, in the presence of linear and circular CPL as in Fig. 1, middle.

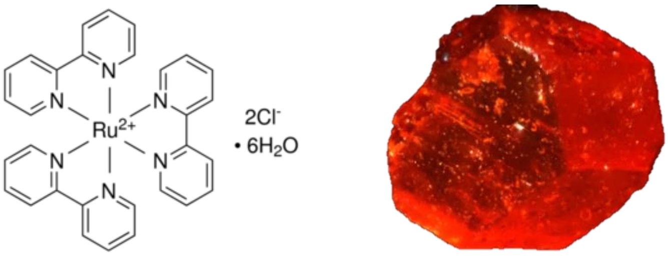

Supplementary figure 3: Chemical structure and a crystal shape example of $[Ru(bpy)_3]Cl_2$. The chemical structure is shown for tris(2,2′-bipyridyl)dichlororuthenium(II) hexahydrate. The crystal image was built based on focus-stacking of a random particle (maximum length: 700 µm).

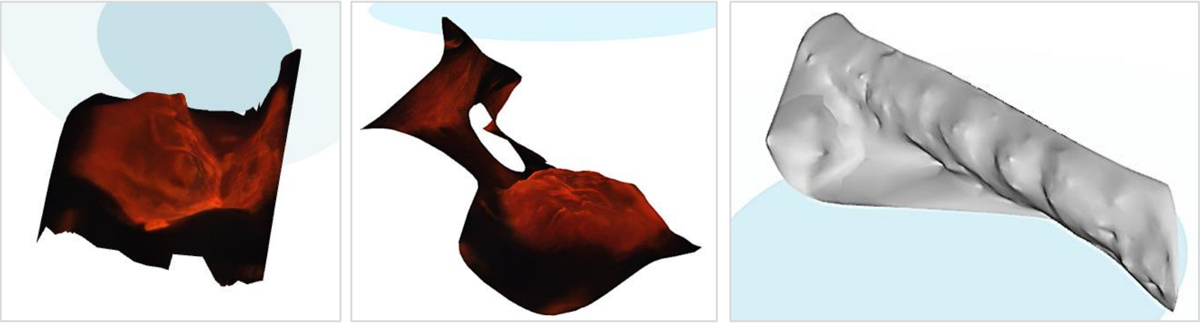

Supplementary figure 4: Challenge examples when using 3D photogrammetry for some datasets and shapes. Left and middle: phosphorescent particle of $[Ru(bpy)_3]Cl_2$ (440 µm) from Fig. 5, c which was reconstructed wrong based on two other 360° dataset images provided. Particle is shown here with the texture fitted. Right: Particles of strontium aluminate placed inside the soda-lime glass capillary tube (length: 1,550 µm), which are the same as in Fig. 4, but based on another 360° dataset. The model shows how the reconstruction may fully or partially curve into a different direction depending on the geometry and images provided. Datasets of models shown here were processed with Autodesk ReMake.

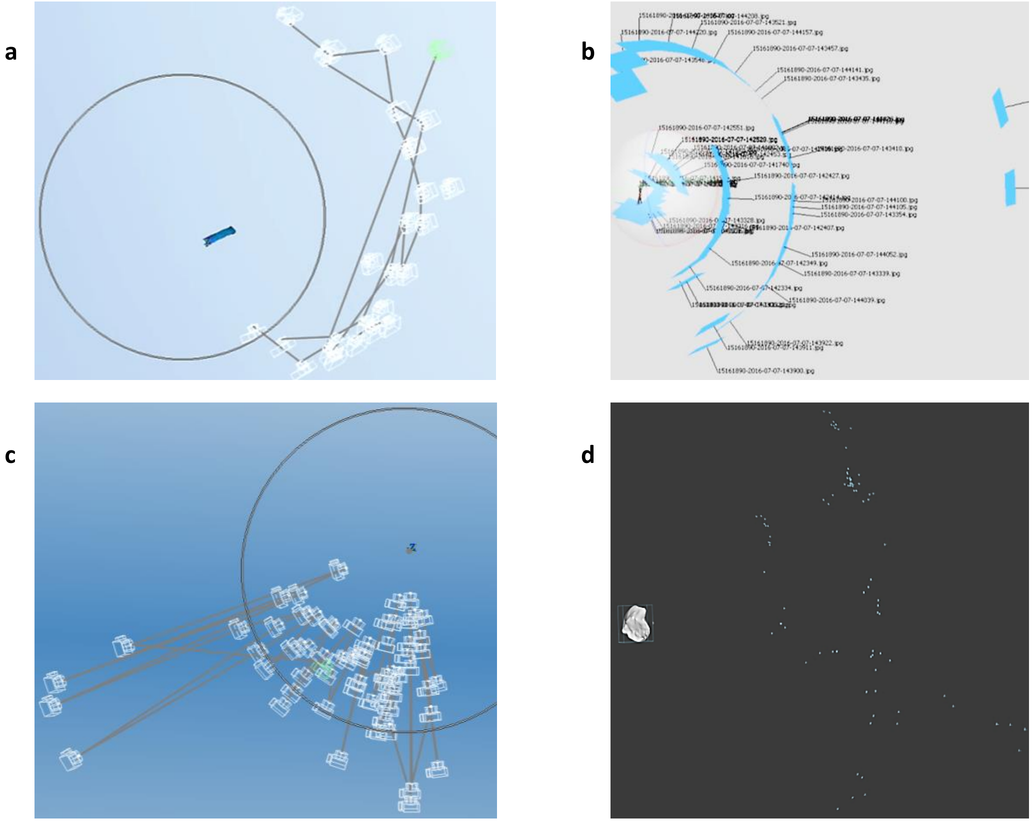

Supplementary figure 5: Individual camera positions as interpreted by 3D photogrammetry programs in different situations. a: The strontium aluminate particles solved in Fig. 4 and processed with Autodesk 123D Catch. b: A similar sample processed with Agisoft Photoscan. c: 360° dataset images processed by Autodesk 123D Catch for a particle where not all images were recognized for the entire 360° rotation reconstruction, but rather for a single side of less than 180°. d: Camera positions of the $[Ru(bpy)_3]Cl_2$ flake particle images (final model shown in Fig. 5, a), as processed by Autodesk ReMake.

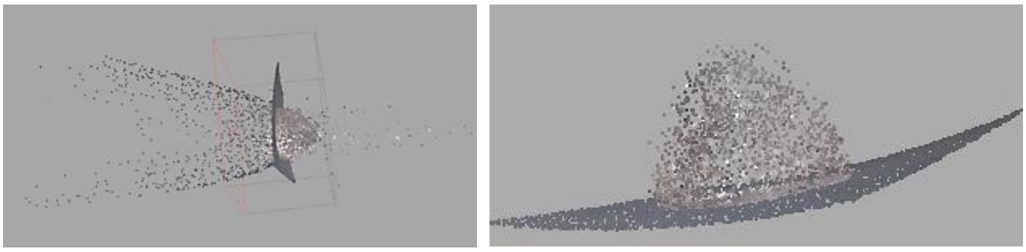

Supplementary figure 6: Difficulty with the 3D reconstruction of highly symmetrical objects such as a sphere. The real image of the sphere shown here is in Fig. 3, a. For attempting to build the 3D model of the sphere, all images were fed into the program (Agisoft PhotoScan), corresponding to 45-60 focal plane images for each of the 20 rotations. Left: Raw result of the dense cloud generated, based on 1,144 cameras corresponding to 7,308 points. Right: Cleaned/refined model with 5,860 points. Single focus-stack images for each of the rotation angles (20 in total) were also used for attempting to build the 3D model, but without success. Program settings used for this figure: 'generic' pair selection, 'high' accuracy, tie point limit of '4,000', key point limit of '40,000', and adaptive camera model fitting 'disabled'.

Macro of functions for analyzing size-shape of dairy particles in ImageJ:

```
run("Enhance Contrast...", "saturated=0.1");
setOption("BlackBackground", true);
run("Smooth");
run("Enhance Contrast...", "saturated=0.1");
run("Make Binary");
run("Smooth");
setOption("BlackBackground", true);
run("Make Binary");
run("Dilate");
run("Despeckle");
run("Remove Outliers...", "radius=15 threshold=50 which=Dark");
run("Remove Outliers...", "radius=15 threshold=50 which=Bright");
run("Smooth");
run("Make Binary");
run("Fill Holes");
run("Set Scale...", "distance=1 known=0.69 pixel=1 unit=um global");
run("Set Measurements...", "area standard min center perimeter bounding shape feret's integrated median area_fraction limit display redirect=None decimal=2");
run("Analyze Particles...", "display exclude summarize record");
```
